# [μ-Bis(diphenyl­phosphan­yl-κ*P*)methane]­deca­carbonyl­tri-μ-hydrido-trirhenium(I)(3 *Re*—*Re*) dichloromethane solvate

**DOI:** 10.1107/S1600536811049312

**Published:** 2011-11-25

**Authors:** Ahmed F. Abdel-Magied, Amrendra K. Singh, Matti Haukka, Ebbe Nordlander

**Affiliations:** a Inorganic Chemistry Research Group, Chemical Physics, Center for Chemistry and Chemical Engineering, Lund University, Box 124, SE-221 00 Lund, Sweden; b Department of Chemistry, University of Eastern Finland, Box 111, FIN-80 101 Joensuu, Finland

## Abstract

In the title compound, [Re_3_(μ-H)_3_(C_25_H_22_P_2_)(CO)_10_]·CH_2_Cl_2_, the three Re atoms form a triangle bearing ten terminal carbonyl groups and three edge-bridging hydrides. The bis­(diphenyl­phosphan­yl)methane ligand bridges two Re atoms. Neglecting the Re—Re inter­actions, each Re atom is in a slightly distorted octa­hedral coordination environment. The dichloro­methane solvent mol­ecule is disordered over two sets of sites with fixed occupancies of 0.6 and 0.4.

## Related literature

For general background to the reaction between rhenium complexes and the bis(diphenylphosphanyl)methane ligand, see: Prest *et al.* (1982 ▶). For related rhenium complexes, see: Adams *et al.* (1993 ▶). For the treatment of the hydride atoms, see: Orpen (1980 ▶). 
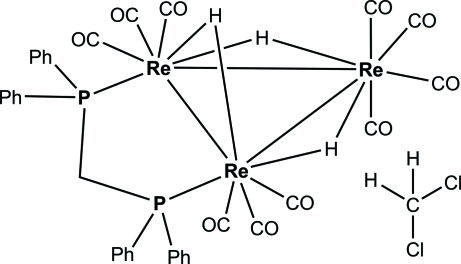


## Experimental

### 

#### Crystal data

[Re_3_H_3_(C_25_H_22_P_2_)(CO)_10_]·CH_2_Cl_2_
*M* *_r_* = 1311.02
Monoclinic, 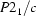

*a* = 16.7907 (6) Å
*b* = 14.5316 (5) Å
*c* = 17.1593 (6) Å
β = 106.445 (1)°
*V* = 4015.5 (2) Å^3^
*Z* = 4
Mo *K*α radiation
μ = 9.29 mm^−1^
*T* = 100 K
0.16 × 0.15 × 0.08 mm

#### Data collection

Bruker Kappa APEXII CCD diffractometer
Absorption correction: multi-scan (*SADABS*; Bruker, 2009 ▶) *T*_min_ = 0.320, *T*_max_ = 0.527
35918 measured reflections
8540 independent reflections
7349 reflections with *I* > 2σ(*I*)
*R*_int_ = 0.031

#### Refinement

*R*[*F* ^2^ > 2σ(*F* ^2^)] = 0.032
*wR*(*F* ^2^) = 0.081
*S* = 1.06
8540 reflections
472 parameters
42 restraints
H-atom parameters constrained
Δρ_max_ = 3.82 e Å^−3^
Δρ_min_ = −2.54 e Å^−3^

### 

Data collection: *APEX2* (Bruker, 2009 ▶); cell refinement: *SAINT* (Bruker, 2009 ▶); data reduction: *SAINT*; program(s) used to solve structure: *SHELXS97* (Sheldrick, 2008 ▶); program(s) used to refine structure: *SHELXL97* (Sheldrick, 2008 ▶); molecular graphics: *ORTEP-3 for Windows* (Farrugia, 1997 ▶); software used to prepare material for publication: *publCIF* (Westrip, 2010 ▶).

## Supplementary Material

Crystal structure: contains datablock(s) I, global. DOI: 10.1107/S1600536811049312/lh5374sup1.cif

Structure factors: contains datablock(s) I. DOI: 10.1107/S1600536811049312/lh5374Isup2.hkl

Supplementary material file. DOI: 10.1107/S1600536811049312/lh5374Isup3.cdx

Additional supplementary materials:  crystallographic information; 3D view; checkCIF report

## Figures and Tables

**Fig. 1.**
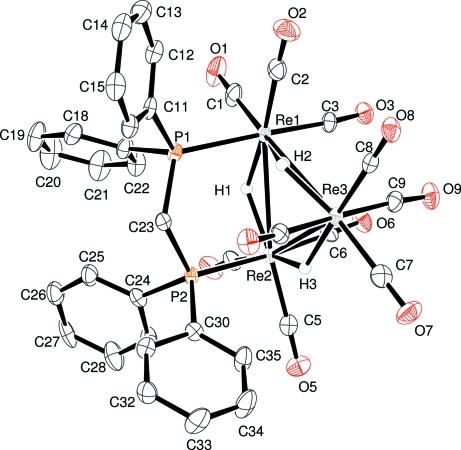
The molecular structure of the title compound, showing 50% probability displacement ellipsoids.

## supplementary crystallographic information

### Comment

The title compound has been reported to form during the reaction between [Re_3_(µ-H)_3_(CO)_12_] and bis[(diphenylphosphino)methane] (dppm) (Prest *et al.*1982); however, its characterization was only based on spectroscopic analysis. We have synthesized this compound during our investigation of similar clusters with diphosphine ligands and the structure of the compound has been established by single-crystal X-ray diffraction. In the title cluster (Fig. 1) the three rhenium atoms form a triangle, bearing ten terminal C≡O groups with three edge-bridging hydrides. The bis[(diphenylphosphino)methane] ligand forms a symmetric bridge over the Re1—Re2 edge. The two triangular edges, Re1—Re3 and Re2—Re3, exhibit bond lengths typical of hydrogen bridged Re—H—Re bonds, 3.2909 (4) and 3.2901 (4) Å, and the third, shorter edge, Re1—Re2 [3.2358 (4) Å], which is doubly bridged by a hydride and the bis[(diphenylphosphino)methane] ligand are comparable with the corresponding interactions in [Re_3_(µ-H)_3_(CO)_10_(µ-dppa)] (dppa = C_2_(PPh_2_)_2_), where the corresponding Re1—Re3 and Re2—Re3 distances are 3.290 (1) and 3.290 (1) Å, respectively, and the doubly bridged Re1—Re2 distance is 3.303 (1) Å (Adams *et al.* 1993). The two Re—P bonds, Re1—P1 and Re2—P2 are very similar, 2.4535 (17) and 2.4592 (16) Å, respectively, and also similar to the corresponding Re—P distances reported for [Re_3_(µ-H)_3_(CO)_10_(µ-dppa)], Re1—P1 and Re2—P2, 2.456 (5) and 2.457 (5) Å, respectively.

### Experimental

The cluster [Re_3_(µ-H)_3_(CO)_11_(NCMe)] (50 mg, 0.055 mmol) and bis[(diphenylphosphino)methane] (42 mg, 0.11 mmol) were stirred for 120 min in dichloromethane (20 ml). A solution of trimethylamine N-oxide (8.2 mg, 0.11 mmol) in dichloromethane (5 ml) was added dropwise during 30 minutes and the reaction was stirred at room temperature until complete conversion had occurred, as judged by spot TLC. The solvent was removed under vacuum and the residue dissolved in a minimum quantity of dichloromethane. The products were then separated by thin layer chromatography on silica using dichloromethane:petroleum ether (1:1) mixture as eluent. The order of elution (decreasing *R*_f_ values) was [Re_2_(CO)_8_(dppm)] (1) and [Re_3_(µ-H)_3_(CO)_10_(dppm)] (2). Final purification was achieved by recrystallization of compound (2) from dichloromethane-hexane.

### Refinement

The dichloromethane of crystallization is disordered over two sites with an occupancy ratio of 0.6/0.4. All C—Cl distances were restrained to be similar and the carbon atoms and chlorine atoms were restrained so that their *U^ij^* components approximate isotropic behavior. Furthermore, all disordered atoms were constrained to have similar anisotropic displacement parameters. Also, the coordinates of C1S and Cl2B were constrained to be the same. The idealized positions of the hydride H atoms were estimated using the XHYDEX program (Orpen, 1980). The hydride H atoms H1H, H2H, and H3H were constrained to ride on Re1, Re2 and Re3, respectively, with *U*_iso_ = 1.5 *U*_eq_(parent Re atom). The hydrogen atoms were positioned geometrically and were also constrained to ride on their parent atoms, with C—H = 0.95 Å, and *U*_iso_ = 1.2 *U*_eq_(parent atom). The highest peak is located 0.08 Å from atom Cl1 and the deepest hole is located 0.32 Å from atom Cl2B.

### Crystal data

**Table:**

[Re_3_H_3_(C_25_H_22_P_2_)(CO)_10_]·CH_2_Cl_2_	*F*(000) = 2448
*M**_r_* = 1311.02	*D*_x_ = 2.169 Mg m^−^^3^
Monoclinic, *P*2_1_/*c*	Mo *K*α radiation, λ = 0.71073 Å
Hall symbol: -P 2ybc	Cell parameters from 9836 reflections
*a* = 16.7907 (6) Å	θ = 2.5–28.3°
*b* = 14.5316 (5) Å	µ = 9.29 mm^−^^1^
*c* = 17.1593 (6) Å	*T* = 100 K
β = 106.445 (1)°	Block, colourless
*V* = 4015.5 (2) Å^3^	0.16 × 0.15 × 0.08 mm
*Z* = 4	

### Data collection

**Table:**

Bruker Kappa APEXII CCD diffractometer	8540 independent reflections
Radiation source: fine-focus sealed tube	7349 reflections with *I* > 2σ(*I*)
horizontally mounted graphite crystal	*R*_int_ = 0.031
Detector resolution: 16 pixels mm^-1^	θ_max_ = 27.0°, θ_min_ = 1.9°
φ scans and ω scans with κ offset	*h* = −19→21
Absorption correction: multi-scan (*SADABS*; Bruker, 2009)	*k* = −18→18
*T*_min_ = 0.320, *T*_max_ = 0.527	*l* = −21→21
35918 measured reflections	

### Refinement

**Table:**

Refinement on *F*^2^	Primary atom site location: structure-invariant direct methods
Least-squares matrix: full	Secondary atom site location: difference Fourier map
*R*[*F*^2^ > 2σ(*F*^2^)] = 0.032	Hydrogen site location: inferred from neighbouring sites
*wR*(*F*^2^) = 0.081	H-atom parameters constrained
*S* = 1.06	*w* = 1/[σ^2^(*F*_o_^2^) + (0.0308*P*)^2^ + 45.3016*P*] where *P* = (*F*_o_^2^ + 2*F*_c_^2^)/3
8540 reflections	(Δ/σ)_max_ = 0.001
472 parameters	Δρ_max_ = 3.82 e Å^−^^3^
42 restraints	Δρ_min_ = −2.54 e Å^−^^3^

### Special details

**Table:**

Geometry. All e.s.d.'s (except the e.s.d. in the dihedral angle between two l.s. planes) are estimated using the full covariance matrix. The cell e.s.d.'s are taken into account individually in the estimation of e.s.d.'s in distances, angles and torsion angles; correlations between e.s.d.'s in cell parameters are only used when they are defined by crystal symmetry. An approximate (isotropic) treatment of cell e.s.d.'s is used for estimating e.s.d.'s involving l.s. planes.
Refinement. Refinement of *F*^2^ against ALL reflections. The weighted *R*-factor *wR* and goodness of fit *S* are based on *F*^2^, conventional *R*-factors *R* are based on *F*, with *F* set to zero for negative *F*^2^. The threshold expression of *F*^2^ > σ(*F*^2^) is used only for calculating *R*-factors(gt) *etc*. and is not relevant to the choice of reflections for refinement. *R*-factors based on *F*^2^ are statistically about twice as large as those based on *F*, and *R*-factors based on ALL data will be even larger.

### Fractional atomic coordinates and isotropic or equivalent isotropic displacement parameters (Å^2^)

**Table:**

	*x*	*y*	*z*	*U*_iso_*/*U*_eq_	Occ. (<1)
Re1	0.351643 (16)	0.492337 (18)	0.253498 (15)	0.01644 (7)	
H1H	0.3064	0.3969	0.1844	0.025*	
Re2	0.347215 (15)	0.278309 (18)	0.200401 (15)	0.01512 (7)	
H2H	0.4030	0.4350	0.3503	0.023*	
Re3	0.485711 (16)	0.347203 (18)	0.367087 (15)	0.01646 (7)	
H3H	0.4244	0.2583	0.2995	0.025*	
P1	0.23187 (10)	0.45699 (12)	0.30346 (10)	0.0171 (3)	
P2	0.24865 (10)	0.24363 (12)	0.27854 (10)	0.0156 (3)	
O1	0.2401 (4)	0.6139 (5)	0.1200 (4)	0.0461 (17)	
O2	0.4131 (4)	0.6613 (4)	0.3622 (4)	0.0428 (15)	
O3	0.4858 (3)	0.5337 (4)	0.1674 (3)	0.0276 (11)	
O4	0.2093 (4)	0.2497 (4)	0.0421 (3)	0.0341 (13)	
O5	0.3835 (3)	0.0763 (4)	0.1708 (3)	0.0317 (12)	
O6	0.4694 (3)	0.3388 (4)	0.1046 (3)	0.0304 (12)	
O7	0.6062 (4)	0.1901 (4)	0.4430 (4)	0.0381 (14)	
O8	0.5898 (3)	0.4864 (4)	0.4905 (3)	0.0275 (12)	
O9	0.6049 (3)	0.3830 (4)	0.2584 (3)	0.0346 (13)	
O10	0.3944 (3)	0.3069 (4)	0.4985 (3)	0.0282 (12)	
C1	0.2817 (5)	0.5669 (6)	0.1700 (5)	0.0308 (18)	
C2	0.3884 (5)	0.5988 (5)	0.3212 (5)	0.0277 (16)	
C3	0.4386 (4)	0.5154 (5)	0.2015 (4)	0.0206 (14)	
C4	0.2602 (4)	0.2650 (5)	0.1007 (4)	0.0229 (15)	
C5	0.3719 (4)	0.1514 (5)	0.1863 (4)	0.0223 (15)	
C6	0.4252 (4)	0.3156 (5)	0.1403 (4)	0.0197 (14)	
C7	0.5611 (5)	0.2486 (5)	0.4131 (4)	0.0270 (16)	
C8	0.5520 (4)	0.4352 (5)	0.4442 (4)	0.0211 (14)	
C9	0.5576 (4)	0.3729 (5)	0.2943 (4)	0.0240 (15)	
C10	0.4252 (4)	0.3192 (5)	0.4483 (4)	0.0209 (14)	
C11	0.2221 (4)	0.5281 (5)	0.3884 (4)	0.0201 (14)	
C12	0.2253 (4)	0.6224 (5)	0.3785 (4)	0.0253 (15)	
H12	0.2265	0.6469	0.3275	0.030*	
C13	0.2270 (5)	0.6815 (6)	0.4424 (5)	0.0304 (17)	
H13	0.2293	0.7461	0.4348	0.036*	
C14	0.2253 (4)	0.6475 (6)	0.5162 (5)	0.0304 (18)	
H14	0.2276	0.6882	0.5602	0.036*	
C15	0.2203 (4)	0.5532 (6)	0.5264 (4)	0.0281 (17)	
H15	0.2182	0.5293	0.5773	0.034*	
C16	0.2182 (4)	0.4935 (5)	0.4629 (4)	0.0227 (15)	
H16	0.2142	0.4291	0.4703	0.027*	
C17	0.1310 (4)	0.4679 (5)	0.2275 (4)	0.0234 (15)	
C18	0.0606 (5)	0.4920 (5)	0.2507 (5)	0.0285 (16)	
H18	0.0658	0.5097	0.3052	0.034*	
C19	−0.0172 (5)	0.4901 (7)	0.1940 (5)	0.041 (2)	
H19	−0.0651	0.5062	0.2099	0.049*	
C20	−0.0250 (5)	0.4648 (8)	0.1147 (5)	0.049 (3)	
H20	−0.0784	0.4633	0.0764	0.058*	
C21	0.0444 (5)	0.4415 (7)	0.0906 (5)	0.043 (2)	
H21	0.0390	0.4243	0.0359	0.051*	
C22	0.1219 (5)	0.4436 (6)	0.1471 (4)	0.0302 (17)	
H22	0.1697	0.4281	0.1306	0.036*	
C23	0.2261 (4)	0.3387 (4)	0.3400 (4)	0.0161 (13)	
H23A	0.2651	0.3339	0.3952	0.019*	
H23B	0.1695	0.3291	0.3453	0.019*	
C24	0.1478 (4)	0.2025 (5)	0.2166 (4)	0.0202 (14)	
C25	0.0718 (4)	0.2421 (6)	0.2124 (5)	0.0289 (17)	
H25	0.0693	0.2940	0.2453	0.035*	
C26	−0.0006 (5)	0.2072 (6)	0.1609 (5)	0.0354 (19)	
H26	−0.0521	0.2358	0.1585	0.042*	
C27	0.0007 (5)	0.1314 (7)	0.1127 (5)	0.037 (2)	
H27	−0.0494	0.1082	0.0771	0.045*	
C28	0.0762 (5)	0.0894 (7)	0.1170 (5)	0.041 (2)	
H28	0.0781	0.0369	0.0846	0.049*	
C29	0.1488 (5)	0.1247 (6)	0.1689 (5)	0.0316 (18)	
H29	0.2002	0.0954	0.1720	0.038*	
C30	0.2760 (4)	0.1528 (5)	0.3552 (4)	0.0183 (13)	
C31	0.2201 (4)	0.1287 (5)	0.3989 (4)	0.0217 (14)	
H31	0.1677	0.1583	0.3874	0.026*	
C32	0.2409 (5)	0.0618 (5)	0.4586 (4)	0.0253 (15)	
H32	0.2030	0.0461	0.4884	0.030*	
C33	0.3170 (5)	0.0177 (6)	0.4750 (5)	0.036 (2)	
H33	0.3316	−0.0277	0.5165	0.043*	
C34	0.3718 (5)	0.0397 (7)	0.4312 (5)	0.041 (2)	
H34	0.4232	0.0081	0.4414	0.049*	
C35	0.3521 (5)	0.1075 (6)	0.3726 (5)	0.0299 (17)	
H35	0.3909	0.1234	0.3438	0.036*	
C1S	−0.0818 (8)	0.2204 (10)	0.3584 (7)	0.199 (3)	0.60
H1S1	−0.1101	0.1945	0.3969	0.238*	0.60
H1S2	−0.0693	0.1696	0.3253	0.238*	0.60
Cl1	0.0123 (9)	0.2785 (10)	0.4128 (11)	0.199 (3)	0.60
Cl2	−0.1495 (9)	0.3089 (10)	0.2913 (7)	0.199 (3)	0.60
C1SB	0.020 (3)	0.244 (3)	0.421 (5)	0.199 (3)	0.40
H1S3	0.0627	0.2072	0.4057	0.238*	0.40
H1S4	0.0250	0.2341	0.4796	0.238*	0.40
Cl1B	0.0272 (15)	0.3638 (16)	0.3988 (11)	0.199 (3)	0.40
Cl2B	−0.0818 (8)	0.2204 (10)	0.3584 (7)	0.199 (3)	0.40

### Atomic displacement parameters (Å^2^)

**Table:**

	*U*^11^	*U*^22^	*U*^33^	*U*^12^	*U*^13^	*U*^23^
Re1	0.01569 (13)	0.02024 (14)	0.01461 (13)	0.00154 (10)	0.00627 (10)	0.00231 (10)
Re2	0.01404 (12)	0.02085 (14)	0.01086 (12)	−0.00069 (10)	0.00418 (9)	−0.00074 (9)
Re3	0.01399 (13)	0.02069 (14)	0.01367 (13)	0.00099 (10)	0.00226 (10)	−0.00083 (10)
P1	0.0156 (8)	0.0220 (9)	0.0142 (8)	0.0041 (7)	0.0053 (6)	0.0028 (6)
P2	0.0126 (8)	0.0214 (9)	0.0127 (8)	0.0003 (6)	0.0034 (6)	0.0007 (6)
O1	0.036 (3)	0.062 (4)	0.047 (4)	0.023 (3)	0.023 (3)	0.036 (3)
O2	0.052 (4)	0.033 (3)	0.052 (4)	−0.013 (3)	0.027 (3)	−0.017 (3)
O3	0.026 (3)	0.034 (3)	0.026 (3)	−0.003 (2)	0.012 (2)	0.000 (2)
O4	0.038 (3)	0.042 (3)	0.018 (3)	−0.013 (3)	−0.001 (2)	0.003 (2)
O5	0.040 (3)	0.030 (3)	0.024 (3)	0.004 (2)	0.006 (2)	−0.004 (2)
O6	0.032 (3)	0.040 (3)	0.026 (3)	−0.011 (2)	0.020 (2)	−0.011 (2)
O7	0.033 (3)	0.030 (3)	0.043 (3)	0.012 (3)	−0.003 (3)	−0.001 (3)
O8	0.019 (3)	0.037 (3)	0.025 (3)	−0.003 (2)	0.004 (2)	−0.012 (2)
O9	0.024 (3)	0.048 (4)	0.036 (3)	−0.004 (3)	0.016 (2)	−0.003 (3)
O10	0.028 (3)	0.039 (3)	0.018 (3)	0.000 (2)	0.006 (2)	0.006 (2)
C1	0.028 (4)	0.038 (5)	0.034 (4)	0.008 (3)	0.021 (3)	0.011 (4)
C2	0.027 (4)	0.031 (4)	0.030 (4)	0.000 (3)	0.016 (3)	0.004 (3)
C3	0.016 (3)	0.029 (4)	0.017 (3)	0.001 (3)	0.004 (3)	−0.001 (3)
C4	0.024 (4)	0.029 (4)	0.015 (3)	−0.006 (3)	0.005 (3)	0.006 (3)
C5	0.021 (3)	0.032 (4)	0.013 (3)	0.000 (3)	0.003 (3)	−0.001 (3)
C6	0.023 (3)	0.020 (3)	0.016 (3)	−0.006 (3)	0.006 (3)	−0.006 (3)
C7	0.026 (4)	0.032 (4)	0.020 (4)	−0.003 (3)	0.002 (3)	−0.006 (3)
C8	0.016 (3)	0.025 (4)	0.022 (3)	0.005 (3)	0.005 (3)	0.002 (3)
C9	0.018 (3)	0.029 (4)	0.021 (3)	0.001 (3)	0.001 (3)	−0.004 (3)
C10	0.016 (3)	0.024 (4)	0.019 (3)	0.000 (3)	−0.001 (3)	0.002 (3)
C11	0.011 (3)	0.031 (4)	0.018 (3)	0.005 (3)	0.004 (3)	0.001 (3)
C12	0.020 (3)	0.035 (4)	0.021 (3)	0.007 (3)	0.006 (3)	0.001 (3)
C13	0.027 (4)	0.034 (4)	0.030 (4)	0.003 (3)	0.008 (3)	−0.006 (3)
C14	0.020 (4)	0.046 (5)	0.023 (4)	0.002 (3)	0.003 (3)	−0.015 (3)
C15	0.021 (4)	0.046 (5)	0.017 (3)	0.000 (3)	0.005 (3)	−0.003 (3)
C16	0.019 (3)	0.032 (4)	0.019 (3)	0.003 (3)	0.008 (3)	0.000 (3)
C17	0.019 (3)	0.030 (4)	0.021 (3)	0.008 (3)	0.004 (3)	0.008 (3)
C18	0.023 (4)	0.032 (4)	0.031 (4)	0.008 (3)	0.007 (3)	0.003 (3)
C19	0.020 (4)	0.063 (6)	0.037 (5)	0.014 (4)	0.004 (3)	0.006 (4)
C20	0.023 (4)	0.085 (8)	0.027 (5)	0.012 (4)	−0.009 (3)	0.012 (5)
C21	0.032 (4)	0.072 (7)	0.019 (4)	0.010 (4)	−0.003 (3)	0.006 (4)
C22	0.024 (4)	0.048 (5)	0.018 (4)	0.003 (3)	0.006 (3)	0.004 (3)
C23	0.013 (3)	0.020 (3)	0.013 (3)	0.000 (2)	0.000 (2)	0.003 (2)
C24	0.020 (3)	0.025 (4)	0.015 (3)	−0.005 (3)	0.003 (3)	0.004 (3)
C25	0.018 (4)	0.033 (4)	0.035 (4)	0.002 (3)	0.006 (3)	0.008 (3)
C26	0.018 (4)	0.042 (5)	0.044 (5)	0.001 (3)	0.005 (3)	0.012 (4)
C27	0.020 (4)	0.064 (6)	0.027 (4)	−0.020 (4)	0.003 (3)	0.003 (4)
C28	0.033 (5)	0.061 (6)	0.031 (4)	−0.018 (4)	0.013 (4)	−0.014 (4)
C29	0.020 (4)	0.044 (5)	0.031 (4)	−0.007 (3)	0.008 (3)	−0.013 (4)
C30	0.018 (3)	0.022 (3)	0.012 (3)	−0.001 (3)	0.000 (3)	0.000 (2)
C31	0.020 (3)	0.027 (4)	0.017 (3)	0.001 (3)	0.003 (3)	−0.002 (3)
C32	0.027 (4)	0.031 (4)	0.021 (3)	−0.001 (3)	0.011 (3)	0.004 (3)
C33	0.042 (5)	0.042 (5)	0.028 (4)	0.020 (4)	0.016 (4)	0.020 (4)
C34	0.032 (4)	0.054 (6)	0.041 (5)	0.023 (4)	0.016 (4)	0.029 (4)
C35	0.022 (4)	0.043 (5)	0.027 (4)	0.008 (3)	0.011 (3)	0.010 (3)
C1S	0.246 (8)	0.210 (8)	0.128 (5)	0.019 (7)	0.033 (5)	−0.037 (5)
Cl1	0.246 (8)	0.210 (8)	0.128 (5)	0.019 (7)	0.033 (5)	−0.037 (5)
Cl2	0.246 (8)	0.210 (8)	0.128 (5)	0.019 (7)	0.033 (5)	−0.037 (5)
C1SB	0.246 (8)	0.210 (8)	0.128 (5)	0.019 (7)	0.033 (5)	−0.037 (5)
Cl1B	0.246 (8)	0.210 (8)	0.128 (5)	0.019 (7)	0.033 (5)	−0.037 (5)
Cl2B	0.246 (8)	0.210 (8)	0.128 (5)	0.019 (7)	0.033 (5)	−0.037 (5)

### Geometric parameters (Å, °)

**Table:**

Re1—C1	1.911 (8)	C15—C16	1.385 (10)
Re1—C2	1.928 (8)	C15—H15	0.9500
Re1—C3	1.945 (7)	C16—H16	0.9500
Re1—P1	2.4535 (17)	C17—C22	1.390 (10)
Re1—Re2	3.2358 (4)	C17—C18	1.393 (10)
Re1—Re3	3.2909 (4)	C18—C19	1.391 (11)
Re1—H1H	1.8430	C18—H18	0.9500
Re1—H2H	1.8410	C19—C20	1.379 (13)
Re2—C4	1.919 (7)	C19—H19	0.9500
Re2—C5	1.920 (8)	C20—C21	1.385 (12)
Re2—C6	1.959 (7)	C20—H20	0.9500
Re2—P2	2.4592 (16)	C21—C22	1.385 (11)
Re2—Re3	3.2901 (4)	C21—H21	0.9500
Re2—H1H	1.8465	C22—H22	0.9500
Re2—H3H	1.8461	C23—H23A	0.9900
Re3—C7	1.927 (8)	C23—H23B	0.9900
Re3—C8	1.947 (7)	C24—C25	1.383 (10)
Re3—C10	1.985 (7)	C24—C29	1.400 (10)
Re3—C9	2.002 (7)	C25—C26	1.381 (11)
Re3—H2H	1.8464	C25—H25	0.9500
Re3—H3H	1.8431	C26—C27	1.382 (13)
P1—C17	1.829 (7)	C26—H26	0.9500
P1—C11	1.831 (7)	C27—C28	1.390 (13)
P1—C23	1.842 (7)	C27—H27	0.9500
P2—C24	1.826 (7)	C28—C29	1.387 (11)
P2—C30	1.827 (7)	C28—H28	0.9500
P2—C23	1.841 (7)	C29—H29	0.9500
O1—C1	1.162 (9)	C30—C35	1.392 (10)
O2—C2	1.153 (9)	C30—C31	1.402 (9)
O3—C3	1.141 (8)	C31—C32	1.384 (10)
O4—C4	1.142 (9)	C31—H31	0.9500
O5—C5	1.153 (9)	C32—C33	1.386 (11)
O6—C6	1.138 (8)	C32—H32	0.9500
O7—C7	1.157 (9)	C33—C34	1.380 (11)
O8—C8	1.142 (8)	C33—H33	0.9500
O9—C9	1.145 (9)	C34—C35	1.378 (11)
O10—C10	1.137 (8)	C34—H34	0.9500
C11—C12	1.384 (11)	C35—H35	0.9500
C11—C16	1.393 (9)	C1S—Cl1	1.801 (13)
C12—C13	1.386 (10)	C1S—Cl2	1.879 (12)
C12—H12	0.9500	C1S—H1S1	0.9900
C13—C14	1.368 (11)	C1S—H1S2	0.9900
C13—H13	0.9500	C1SB—Cl1B	1.790 (16)
C14—C15	1.388 (12)	C1SB—H1S3	0.9900
C14—H14	0.9500	C1SB—H1S4	0.9900
			
C1—Re1—C2	91.2 (4)	O2—C2—Re1	177.7 (7)
C1—Re1—C3	86.6 (3)	O3—C3—Re1	174.9 (6)
C2—Re1—C3	89.1 (3)	O4—C4—Re2	174.5 (7)
C1—Re1—P1	89.4 (2)	O5—C5—Re2	174.1 (6)
C2—Re1—P1	96.4 (2)	O6—C6—Re2	178.6 (7)
C3—Re1—P1	173.3 (2)	O7—C7—Re3	177.9 (7)
C1—Re1—Re2	111.9 (3)	O8—C8—Re3	178.5 (6)
C2—Re1—Re2	156.8 (2)	O9—C9—Re3	173.1 (6)
C3—Re1—Re2	89.8 (2)	O10—C10—Re3	175.3 (6)
P1—Re1—Re2	86.64 (4)	C12—C11—C16	118.8 (7)
C1—Re1—Re3	168.3 (2)	C12—C11—P1	116.5 (5)
C2—Re1—Re3	96.3 (2)	C16—C11—P1	124.4 (6)
C3—Re1—Re3	84.6 (2)	C11—C12—C13	120.7 (7)
P1—Re1—Re3	98.61 (4)	C11—C12—H12	119.6
Re2—Re1—Re3	60.536 (8)	C13—C12—H12	119.6
C1—Re1—H1H	83.7	C14—C13—C12	120.5 (8)
C2—Re1—H1H	174.1	C14—C13—H13	119.8
C3—Re1—H1H	93.5	C12—C13—H13	119.8
P1—Re1—H1H	80.7	C13—C14—C15	119.4 (7)
Re2—Re1—H1H	28.7	C13—C14—H14	120.3
Re3—Re1—H1H	89.3	C15—C14—H14	120.3
C1—Re1—H2H	164.8	C16—C15—C14	120.6 (7)
C2—Re1—H2H	80.3	C16—C15—H15	119.7
C3—Re1—H2H	105.7	C14—C15—H15	119.7
P1—Re1—H2H	79.1	C15—C16—C11	119.9 (7)
Re2—Re1—H2H	77.7	C15—C16—H16	120.0
Re3—Re1—H2H	26.9	C11—C16—H16	120.0
H1H—Re1—H2H	104.0	C22—C17—C18	118.9 (7)
C4—Re2—C5	85.9 (3)	C22—C17—P1	120.3 (5)
C4—Re2—C6	90.7 (3)	C18—C17—P1	120.5 (6)
C5—Re2—C6	89.9 (3)	C19—C18—C17	119.9 (7)
C4—Re2—P2	90.3 (2)	C19—C18—H18	120.0
C5—Re2—P2	94.3 (2)	C17—C18—H18	120.0
C6—Re2—P2	175.7 (2)	C20—C19—C18	120.3 (8)
C4—Re2—Re1	107.4 (2)	C20—C19—H19	119.8
C5—Re2—Re1	165.9 (2)	C18—C19—H19	119.8
C6—Re2—Re1	85.2 (2)	C19—C20—C21	120.4 (8)
P2—Re2—Re1	90.51 (4)	C19—C20—H20	119.8
C4—Re2—Re3	167.9 (2)	C21—C20—H20	119.8
C5—Re2—Re3	106.1 (2)	C20—C21—C22	119.3 (8)
C6—Re2—Re3	87.73 (19)	C20—C21—H21	120.4
P2—Re2—Re3	90.43 (4)	C22—C21—H21	120.4
Re1—Re2—Re3	60.561 (8)	C21—C22—C17	121.1 (7)
C4—Re2—H1H	78.7	C21—C22—H22	119.4
C5—Re2—H1H	164.1	C17—C22—H22	119.4
C6—Re2—H1H	86.5	P2—C23—P1	117.8 (3)
P2—Re2—H1H	89.6	P2—C23—H23A	107.9
Re1—Re2—H1H	28.7	P1—C23—H23A	107.9
Re3—Re2—H1H	89.2	P2—C23—H23B	107.9
C4—Re2—H3H	164.6	P1—C23—H23B	107.9
C5—Re2—H3H	81.1	H23A—C23—H23B	107.2
C6—Re2—H3H	97.4	C25—C24—C29	118.0 (7)
P2—Re2—H3H	82.6	C25—C24—P2	125.8 (6)
Re1—Re2—H3H	86.4	C29—C24—P2	116.2 (5)
Re3—Re2—H3H	26.9	C26—C25—C24	120.8 (8)
H1H—Re2—H3H	114.7	C26—C25—H25	119.6
C7—Re3—C8	91.6 (3)	C24—C25—H25	119.6
C7—Re3—C10	88.2 (3)	C25—C26—C27	121.1 (8)
C8—Re3—C10	88.0 (3)	C25—C26—H26	119.5
C7—Re3—C9	87.2 (3)	C27—C26—H26	119.5
C8—Re3—C9	88.4 (3)	C26—C27—C28	119.1 (7)
C10—Re3—C9	174.1 (3)	C26—C27—H27	120.5
C7—Re3—Re2	110.5 (2)	C28—C27—H27	120.5
C8—Re3—Re2	156.6 (2)	C29—C28—C27	119.7 (8)
C10—Re3—Re2	99.9 (2)	C29—C28—H28	120.2
C9—Re3—Re2	85.2 (2)	C27—C28—H28	120.2
C7—Re3—Re1	168.3 (2)	C28—C29—C24	121.3 (8)
C8—Re3—Re1	98.3 (2)	C28—C29—H29	119.3
C10—Re3—Re1	98.4 (2)	C24—C29—H29	119.3
C9—Re3—Re1	86.7 (2)	C35—C30—C31	118.6 (6)
Re2—Re3—Re1	58.903 (8)	C35—C30—P2	121.7 (5)
C7—Re3—H2H	162.8	C31—C30—P2	119.7 (5)
C8—Re3—H2H	85.0	C32—C31—C30	120.4 (6)
C10—Re3—H2H	74.9	C32—C31—H31	119.8
C9—Re3—H2H	109.5	C30—C31—H31	119.8
Re2—Re3—H2H	76.1	C31—C32—C33	120.0 (7)
Re1—Re3—H2H	26.8	C31—C32—H32	120.0
C7—Re3—H3H	85.5	C33—C32—H32	120.0
C8—Re3—H3H	176.3	C34—C33—C32	120.0 (7)
C10—Re3—H3H	89.6	C34—C33—H33	120.0
C9—Re3—H3H	93.8	C32—C33—H33	120.0
Re2—Re3—H3H	26.9	C35—C34—C33	120.3 (7)
Re1—Re3—H3H	84.8	C35—C34—H34	119.9
H2H—Re3—H3H	97.2	C33—C34—H34	119.9
C17—P1—C11	104.1 (3)	C34—C35—C30	120.7 (7)
C17—P1—C23	101.3 (3)	C34—C35—H35	119.6
C11—P1—C23	103.3 (3)	C30—C35—H35	119.6
C17—P1—Re1	114.7 (2)	Cl1—C1S—Cl2	106.6 (10)
C11—P1—Re1	115.3 (2)	Cl1—C1S—H1S1	110.4
C23—P1—Re1	116.2 (2)	Cl2—C1S—H1S1	110.4
C24—P2—C30	100.7 (3)	Cl1—C1S—H1S2	110.4
C24—P2—C23	105.7 (3)	Cl2—C1S—H1S2	110.4
C30—P2—C23	100.4 (3)	H1S1—C1S—H1S2	108.6
C24—P2—Re2	114.0 (2)	Cl1B—C1SB—H1S3	111.8
C30—P2—Re2	118.0 (2)	Cl1B—C1SB—H1S4	111.8
C23—P2—Re2	116.0 (2)	H1S3—C1SB—H1S4	109.6
O1—C1—Re1	178.6 (9)		
